# Transcriptional heterogeneity between primary adult grey and white matter astrocytes underlie differences in modulation of *in vitro* myelination

**DOI:** 10.1186/s12974-020-02045-3

**Published:** 2020-12-11

**Authors:** Inge L. Werkman, Marissa L. Dubbelaar, Pieter van der Vlies, Jelkje J. de Boer-Bergsma, Bart J. L. Eggen, Wia Baron

**Affiliations:** 1 Biomedical Sciences of Cells & Systems, Section Molecular Neurobiology, University of Groningen, University Medical Center Groningen, A. Deusinglaan 1, 9713 AV Groningen, the Netherlands; 2 Department of Genetics, University of Groningen, University Medical Center Groningen, Groningen, the Netherlands

**Keywords:** Astrocytes, Multiple sclerosis, Myelination, Oligodendrocyte, Region

## Abstract

**Background:**

Multiple sclerosis (MS) is an inflammation-mediated demyelinating disease of the central nervous system that eventually results in secondary axonal degeneration due to remyelination failure. Successful remyelination is orchestrated by astrocytes (ASTRs) and requires sequential activation, recruitment, and maturation of oligodendrocyte progenitor cells (OPCs). In both MS and experimental models, remyelination is more robust in grey matter (GM) than white matter (WM), which is likely related to local differences between GM and WM lesions. Here, we investigated whether adult gmASTRs and wmASTRs per se and in response to MS relevant Toll-like receptor (TLR) activation differently modulate myelination.

**Methods:**

Differences in modulation of myelination between adult gmASTRs and wmASTRs were examined using an in vitro myelinating system that relies on a feeding layer of ASTRs. Transcriptional profiling and weighted gene co-expression network analysis were used to analyze differentially expressed genes and gene networks. Potential differential modulation of OPC proliferation and maturation by untreated adult gmASTRs and wmASTRs and in response to TLR3 and TLR4 agonists were assessed.

**Results:**

Our data reveal that adult wmASTRs are less supportive to in vitro myelination than gmASTRs. WmASTRs more abundantly express reactive ASTR genes and genes of a neurotoxic subtype of ASTRs, while gmASTRs have more neuro-reparative transcripts. We identified a gene network module containing cholesterol biosynthesis enzyme genes that positively correlated with gmASTRs, and a network module containing extracellular matrix-related genes that positively correlated with wmASTRs. Adult wmASTRs and gmASTRs responding to TLR3 agonist Poly(I:C) distinctly modulate OPC behavior, while exposure to TLR4 agonist LPS of both gmASTRs and wmASTRs results in a prominent decrease in myelin membrane formation.

**Conclusions:**

Primary adult gmASTRs and wmASTRs are heterogeneous at the transcriptional level, differed in their support of in vitro myelination, and their pre-existing phenotype determined TLR3 agonist responses. These findings point to a role of ASTR heterogeneity in regional differences in remyelination efficiency between GM and WM lesions.

**Supplementary Information:**

The online version contains supplementary material available at 10.1186/s12974-020-02045-3.

## Background

Remyelination is a natural regenerative process that occurs after demyelination, to form new myelin membranes around denuded axons. In the central nervous system (CNS), remyelination is executed by oligodendrocytes (OLGs) and involves activation, recruitment, and differentiation of oligodendrocyte progenitor cells (OPCs) into mature, myelinating OLGs [1]. Remyelination is orchestrated by transient signaling of reactive astrocytes (ASTRs) [2, 3]. Similar as during CNS development [4], ASTRs support oligodendrocytes and myelin membrane formation by extracellular matrix (ECM) remodeling [5, 6] and the supply of fatty acids and cholesterol [4, 7, 8]. Upon demyelination, ASTR reactivity is induced by a variety of inflammatory mediators such as Toll-like receptor (TLR)ligands [9–13] as well as by myelin debris, and includes changes in ASTR morphology, gene expression, and function [14–19]. ASTR reactivity can be beneficial or detrimental for remyelination [2, 12, 20, 21], as ASTR responses toward injury vary and depend on the type of injury. In this regard, two subtypes of injury-dependent reactive ASTRs have been described; neuro-inflammation-induced A1-ASTRs and ischemia-induced A2-ASTRs [14, 22]. A1-ASTRs possess a neurotoxic phenotype and secrete factors that inhibit OPC proliferation, migration, and differentiation, and that are toxic to mature OLGs [22]. By contrast, A2-ASTRs appear more neuroprotective and more supportive toward repair [22]. Although the classification of ASTR reactivity into A1-ASTRs and A2-ASTRs is very useful as a concept, it is clear that additional injury-dependent astrocyte reactivity states exist [23]. Also at homeostatic conditions, ASTRs are not uniform, and multiple studies have described ASTRs diversity based on their morphology, gene expression, and function [22, 24–30].

Historically, ASTRs are divided into two groups; fibrous ASTRs that mainly reside in the white matter (WM) and the morphologically more complex protoplasmic ASTRs that are present in the grey matter (GM) [27, 31, 32]. Multiple single-nucleus RNA sequencing studies identify two to three clear distinct groups of ASTRs dispersed throughout adult GM and WM [29, 33–35], while morphological and functional studies identify up to nine different groups of ASTRs in rodents [25, 36]. ASTR reactivity is also region-dependent, i.e., upon cuprizone-induced demyelination, ASTRs reactivity is evident earlier in the cortex, a GM area, than the corpus callosum, a WM area, but ASTR reactivity is more extensive in ASTRs of the corpus callosum [19, 37–39]. Furthermore, demyelination is delayed in the cortex compared to the corpus callosum [38], and remyelination occurs faster in GM than in WM areas [37]. Also, in the chronic demyelinating disease multiple sclerosis (MS), remyelination is more efficient in GM than in WM [40, 41], but ultimately fails in both areas, contributing to disease progression [1, 42, 43]. As in toxin-induced demyelination rodent models, ASTR reactivity differs between GM and WM MS lesions. Hypertrophic ASTRs form a glial scar in and around inflammatory WM lesions, but not around lesions in the GM [12, 15, 44, 45]. This difference in ASTR reactivity may contribute to the more efficient remyelination in GM MS lesions [3, 37, 41, 46]. In addition, neurotoxic A1-ASTRs are present in WM MS lesions [22], while the presence of A1-ASTRs in GM MS lesions has not been analyzed yet. It is currently not clear whether intrinsic differences between gmASTRs and wmASTRs contribute to regional differences in ASTR reactivity and/or whether they differently respond to the same type of injury and inflammation. By performing transcriptional profiling and the use of an in vitro myelinating system that depend on ASTRs, we addressed here whether adult gmASTRs and wmASTRs are different cell populations that distinctly modulate myelination. In addition, as innate TLR-mediated signaling plays a role in MS pathology [47–50], we assessed whether gmASTRs and wmASTRs display a differential response to TLR3 and TLR4 agonists. Our findings revealed that adult wmASTRs and gmASTRs display transcriptional heterogeneity and that wmASTRs are less supportive to in vitro myelination.

## Methods

### Primary cell cultures

#### Adult astrocytes

Adult astrocytes (ASTRs) were isolated from young adult female Wistar rats brains via a non-shake off procedure as described [51], with minor modifications. To obtain gmASTRs, the cerebral cortices were dissected and meninges removed. Of the residual non-cortex tissue, the amygdala was discarded and the remaining non-cortical regions (WM tracts including corpus callosum, mixed GM and WM tracts, including hippocampus and thalamus, and deep GM parts, including basal ganglia) were kept in HBSS. Although the dissected non-cortical ASTRs contain gmASTRs, they mainly consist of wmASTRs and were therefore referred to as wmASTRs. Brain tissue was kept in Hank’s balanced salt solution (HBSS, Life Technologies) and mechanically dissected using scissors. Then, 0.05% trypsin (Sigma) and 0.003% DNase (Roche) in 1 mL HBSS was added and incubated for 15 min at 37 °C, followed by centrifugation for 7 min at 400 g. The pellet was resuspended in papain digestion mix [24 μg/mL l-cysteine (Sigma), 40 μg/mL DNase I, 30 U/mL papain from papaya latex (Sigma) in MEM (Life Technologies)] incubated for 15 min at 37 °C. The digestion was stopped using OVO [40 μg/mL DNase I, 1 mg/mL soybean trypsin inhibitor (Sigma), 50 μg/mL bovine serum albumin (BSA, Sigma) in Leibowitz L-15 medium (Sigma)] for 6 min at room temperature. The cell suspension was centrifuged for 7 min at 400 g and resuspended in 15 mL HBSS. Hereafter, the cells were settled for 30 min before the upper layer was separated from the lower cell fraction. The cell fractions were diluted in 15 mL HBSS and centrifuged for 7 min at 400 g. Cell pellets were resuspended in AA+ medium [10% fetal bovine serum (FBS), 15 mM Hepes (Gibco), 0.04% gentamicin (Life Technologies), 14.3 mM NaHCO_3_ (Merck), 100 U/mL penicillin, 100 μg/mL streptomycin, 4 mM l-glutamine]. The cells were cultured for 20 days at 37 °C in tissue flasks (Nunc T80; Thermo Fisher Scientific) that were coated with poly-l-lysine (PLL, 5 μg/mL, Sigma). Medium exchange occurred once every 3–4 days. The cells were passaged at least once using trypsin and used for experiments after 2–3 weeks, or collected in RNA protect (Qiagen) for 3′-RNA sequencing. To obtain ASTR-conditioned medium (ACM), ASTRs were cultured for 24 h either untreated or in the presence of 50 μg/mL TLR3 agonist polyinosine-polycytidylic acid (Poly(I:C), Sigma) or 200 ng/mL TLR4 agonist lipopolysaccharide (LPS, Sigma), after which cells were washed and cultured for another 24 h in defined SATO medium [5 μg/mL bovine insulin (Sigma), 50 μg/mL human holo-transferrin (Sigma), 100 μg/mL BSA fraction V (Sigma), 62 ng/mL progesterone (Sigma), 16 μg/mL putrescine (Sigma), 5 ng/mL sodium selenite (Sigma), 400 ng/mL T3 (Sigma), 400 ng/mL T4 (Sigma), 4 mM l-glutamine, 100 U/mL penicillin and streptomycin]. Hereafter, ACM was collected and filtered using a 0.45 μm filter, to remove cell debris. To obtain ASTR extracellular matrix (ECM), ASTRs were cultured for 48 h either untreated or in the presence of Poly(I:C) or LPS, after which cells were lysed by water (two times 1 h, cell lysis was checked by a microscope) and ECM was scraped in sterile PBS containing a cocktail of protease inhibitors (Complete, Roche). Notably, adult gmASTRs and wmASTRs were always generated at the same time from the same animal and cultured at similar conditions.

#### Oligodendrocyte progenitor cells

Oligodendrocyte progenitor cells (OPCs) were isolated from mixed glia cultures of the cortex of newborn rat forebrains using a shake-off procedure as described [52–54]. Briefly, contaminating microglia were removed from the flask by a pre-shake on an orbital shaker (Innova 4000, New Brunswick) at 150 rpm for 1 h at 37 °C and OPCs were obtained after a shake at 240 rpm for 20–22 h at 37 °C. The detached OPCs were further purified by differential adhesion on non-tissue dishes [54]. The enriched OPC cultures contained 95–97% OPCs (Olig2-positive), 1–3% astrocytes (GFAP-positive), and less than 1% microglia (IB4-positive) and neurons (TuJ1-positive). The OPCs were cultured on PLL-coated 13-mm coverslips (35,000 cells/coverslip) or on ECM-coated 8-well Permanox chamber slides (8 μg of ECM/chamber 28,000 cells/chamber). For proliferation, OPCs were cultured for 24 h in defined SATO medium containing 10 ng/mL platelet-derived growth factor-AA (PDGF-AA, Peprotech) and 10 ng/mL fibroblast growth factor-2 (FGF2, Peprotech) in the presence or absence of ACM (diluted 1:1). For other assays, cells were cultured for 2 days in SATO medium supplemented with PDGF-AA and FGF2, followed by differentiation upon growth factor withdrawal and culturing for 6 days in SATO supplemented with 0.5% FBS. Cells were cultured in the presence or absence of ACM (diluted 1:1) or on ECM coatings, after which differentiation, myelin membrane formation, and metabolic activity were determined.

#### Spinal cord cultures

Myelinating spinal cord cultures that rely on a monolayer of feeding ASTRs were generated from 17-day-old Wistar rat embryos as described with minor modifications [55]. Meninges were removed from the dissected spinal cord, and mechanical dissociation of the tissue was performed in ΜΕΜ. Hereafter, the tissue was enzymatically digested with a mixture of trypsin (2.5%, Sigma) and liberase DH (2.5 mg/mL, Roche) for 20 min at 37 °C. The enzymatic reaction was stopped by addition of Soybean trypsin inhibitor solution [0.52 mg/mL soybean trypsin inhibitor (Sigma), 40 μg/mL DNase (Roche), and 3 mg/mL BSA fraction V in Leibovitz’s L15 medium]. After centrifugation for 7 min at 220 g, cells were resuspended in plating medium consisting of 50% DMEM (1500 mg/L glucose, Gibco), 25% horse serum (Invitrogen), 25% HBSS with calcium and magnesium (Gibco), and 2 mM l-glutamine (Invitrogen). The total spinal cord cell suspension was plated at a density of 200,000 cells/well in a 24-wells containing a 2-day-old confluent feeding layer of adult gmASTRs or wmASTRs (120,000 cells/24 well) in 500 μL plating medium. When cells were attached, 500 μL growth medium [DMEM (4500 mg/L glucose, Gibco) supplemented with 5 mg/mL holotransferin (Sigma), 20 mM putrescine (Sigma), 4 μM progesterone (Sigma), 6 μM selenium (Sigma), 10 ng/mL biotin (Sigma), 50 nM hydrocortisone (Sigma), and 10 μg/mL insulin (Sigma)] was added. Every 2–3 days, half of the medium was replaced with new growth medium. Insulin was omitted from growth medium after 12 days in culture and cultures were analyzed at 26–30 days in culture.

### Gene expression and WGCNA

RNA was isolated using the RNeasy Plus Micro Kit (QIAGEN) and RNA quality was assessed using a BioRad Experion Highsense RNA kit according to manufacturer’s instructions. All RIN-values were higher than 7.9. Library preparation was performed with the Lexogen QuantSeq 3′mRNA-Seq Library Prep kit for Ilumina according to manufacturer’s instructions. All samples were pooled and sequenced on the Illumina NexSeq 500. Data was processed using the pipelines that were provided by MOLGENIS (https://github.com/molgenis). Quality control of the data was performed on the raw fastq files with fastQC (0.11.3). Next, HiSat (0.1.5) was used for alignment of the sequenced reads against the Rattus norvegicus genome (38.82) allowing 2 mismatches, and the aligned data was sorted with samtools (1.2). Finally, HTSeq (0.6.1p1) was used to quantify the data using the parameters: --stranded=no and --mode=union. High and low expressed genes were distinguished using the Data-adaptive flag method for RNA-sequencing (DAFS). Normalization and processing of the raw reads were performed with EdgeR (3.20.9). To determine differentially expressed genes, an absolute logarithm of the fold change (logFC) of more than 2 and an FDR < 0.01 were used. Volcano plots were made using the ggplot2 package. The DAFS filtered genes were used as input to generate a signed network (with a soft power of 10) with the topological overlap matrix (TOM) function (TOMsimilarity). Cutting of the hierarchical clustered TOM with the function cutreeDynamic with a minimum cluster size of 100 and cutHeight 0.99 was used to determine the different modules. Afterward, similar gene expression modules were merged with mergeCloseModules (cutHeight 0.25). In the end, userListEnrichment was used to identify possible traits of the identified modules where the grey lists were omitted, with enabling of the parameters: useBrainLists, useBloodAtlases, useStemCellLists, useBrainRegionMarkers, usePalazzoloWang, useImmunePathwayLists. For Gene Ontology and Gene-Concept Network visualization, Clusterprofiler with the enrichplot package were used. All analyses were performed in R.

### Western blotting

Cells were scraped in 500 μL of lysis buffer (1% Triton X-100, 50 mM Tris-HCl, 150 mM NaCl, 5 mM EDTA, and protease inhibitor cocktail). The total protein concentration was determined using a BioRad DC-protein assay and BSA as standard according to manufacturer’s instructions. Cell lysates (20 μg) were loaded onto a 7.5% SDS-polyacrylamide gel. After gel electrophoresis, proteins were transferred onto polyvinylidene fluoride (PVDF) membranes (Immobilon-FL, Merck Millipore) by wet transfer. PVDF membranes were blocked for 1 h with Odyssey blocking buffer (Li-Cor Biosciences), and incubated overnight with primary antibodies against GFAP (anti-GFAP, polyclonal, 1:5000, Dako, Z033430) at 4 °C. After washing with PBS containing 1% Tween-20, membranes were incubated with IRDye-conjugated secondary antibodies (Li-Cor Biosciences, Lincoln; 1:3000) for 1 h. As loading control, β-actin (monoclonal mouse anti-β-actin, 1:2000, Sigma, A5441) was used. The membranes were scanned using the Odyssey Imaging System (Li-Cor), and analyzed by densitometry using FIJI ImageJ (NIH).

### Immunocytochemistry

#### Primary cells

Live and fixed immunostainings were performed as described [54]. For live cell immunolabelling, non-specific antibody binding was blocked with 4% BSA for 10 min at 4 °C, followed by incubation of the cells with A2B5 antibody (1:5, kind gift of Dr. Thijs Lopez-Cardozo, Utrecht, the Netherlands) for 30 min at 4 °C. Cells were rinsed twice with PBS and incubated with appropriate Alexa-conjugated antibody (1:500, Millipore) for 25 min at 4 °C. After two washes with PBS, cells were fixed with 4% paraformaldehyde (PFA) in PBS for 20 min at room temperature and incubated for 15 min with 1 μg/mL DAPI (Sigma) to counterstain nuclei. For staining of internal antigens, PFA-fixed cells were permeabilized with ice-cold methanol for 10 min. Non-specific antibody binding was blocked with 4% BSA for 30 min after which cells were incubated with either anti-Ki67 (1 μg/mL, Abcam, ab15580) or anti-myelin basic protein (MBP, 1:250, Serotec, MCA409S) antibodies at room temperature. Cells were washed three times with PBS before the appropriate Alexa-conjugated antibodies (1:500, Millipore) were added together with 1 μg/mL DAPI for 30 min at room temperature. After washing with PBS, cells were mounted with mounting medium (Dako). Cells were analyzed using a conventional immunofluorescence microscope (Leica DMI 6000 B) equipped with Leica Application Suite Advanced Fluorescence software. In each independent experiment, for each condition, 15 random images were acquired resulting in the analysis of 150–250 cells per condition. Proliferation was defined as the percentage Ki67-positive of A2B5-positive cells, differentiation as the percentage of MBP-positive cells of DAPI-stained cells, and myelin membrane formation as the percentage of MBP-positive cells that elaborate myelin membranes irrespective of the extent of the membrane. Notably, myelin membranes are membranous structures spread between cellular processes.

#### Spinal cord cultures

Spinal cord cultures were fixed with 4% PFA for 30 min, followed by blocking and permeabilization with 0.1% Triton X-100 in 4% BSA in PBS for 45 min. Cells were washed thrice with PBS and incubated with anti-MBP antibody (1:250) and anti-neurofilament-H (NF, polyclonal chicken anti-neurofilament, 1:5000, EnCor Biotechnology Inc., 2796-7) for 90 min at room temperature. After washing twice with PBS, cultures were incubated with appropriate FITC- or TRITC-conjugated secondary antibodies (1:50, Jackson Immunolaboratories) combined with DAPI for 45 min at room temperature. Coverslips were mounted and cultures were analyzed by confocal microscopy (SP8 AOBS Microscope, Leica Microsystems) using Leica Confocal Software. The percentage of myelinated axons was calculated in ImageJ as an area in pixels in each image occupied by both myelin and axons dived by the axonal density as described [56, 57]. In each experiment, 5 images per coverslip and 2 coverslips per condition were analyzed.

### Metabolic activity and cytotoxicity

Metabolic activity of cells was assessed by 3-(4,5-dimethyl-2-thiazolyl)-2,5-diphenyl-2H-tetrazolium bromide (MTT; Sigma)-reduction, while cytotoxicity was analyzed with a lactate dehydrogenase (LDH; Roche) assay. For the MTT-reduction assay, 500 μg/mL MTT was added to each well and left to incubate for 4 h at 37 °C. Cells were resuspended in dimethylsulfoxide and absorption was measured at 570 nm. Data represent values relative to control. LDH assays were performed according to manufacturer’s instructions using the medium of the cells from the MTT-reduction assay and related to LDH in medium of lysed untreated cells.

### Statistics

Data are expressed as mean ± standard error of the mean (SEM) for at least three independent experiments. When relative values of groups were compared to NCM, no ECM, control ACM, or control ECM coatings, statistical analysis was performed with a one-sample *t* test by setting the untreated control values at 1 in each independent experiment. In all cases, *p* values of < 0.05, < 0.01, and < 0.001 were considered significant and indicated with *, **, and ***, respectively. When values between two groups of the same cell culture preparations (wmACM versus gmACM and wmECM versus gmECM) were compared, statistical significance was assessed using a paired two-sided *t* test. When values between treatment of gmASTRs or wmASTRs on OPC behavior were compared, an unpaired two-sided *t* test was used to assess statistical significance. Here, *p* values of < 0.05, < 0.01, and <v0.001 were considered significant and indicated with ^#^, ^##^, and ^###^, respectively. Statistics were performed using GraphPad Prism 6.0. In heatmaps of RNAseq data FDR-values of < 0.05, < 0.01, and < 0.001 were considered significant and indicated with *, **, ***.

## Results

### Primary adult wmASTRs and gmASTRs are transcriptionally heterogeneous

**Fig. 1:**
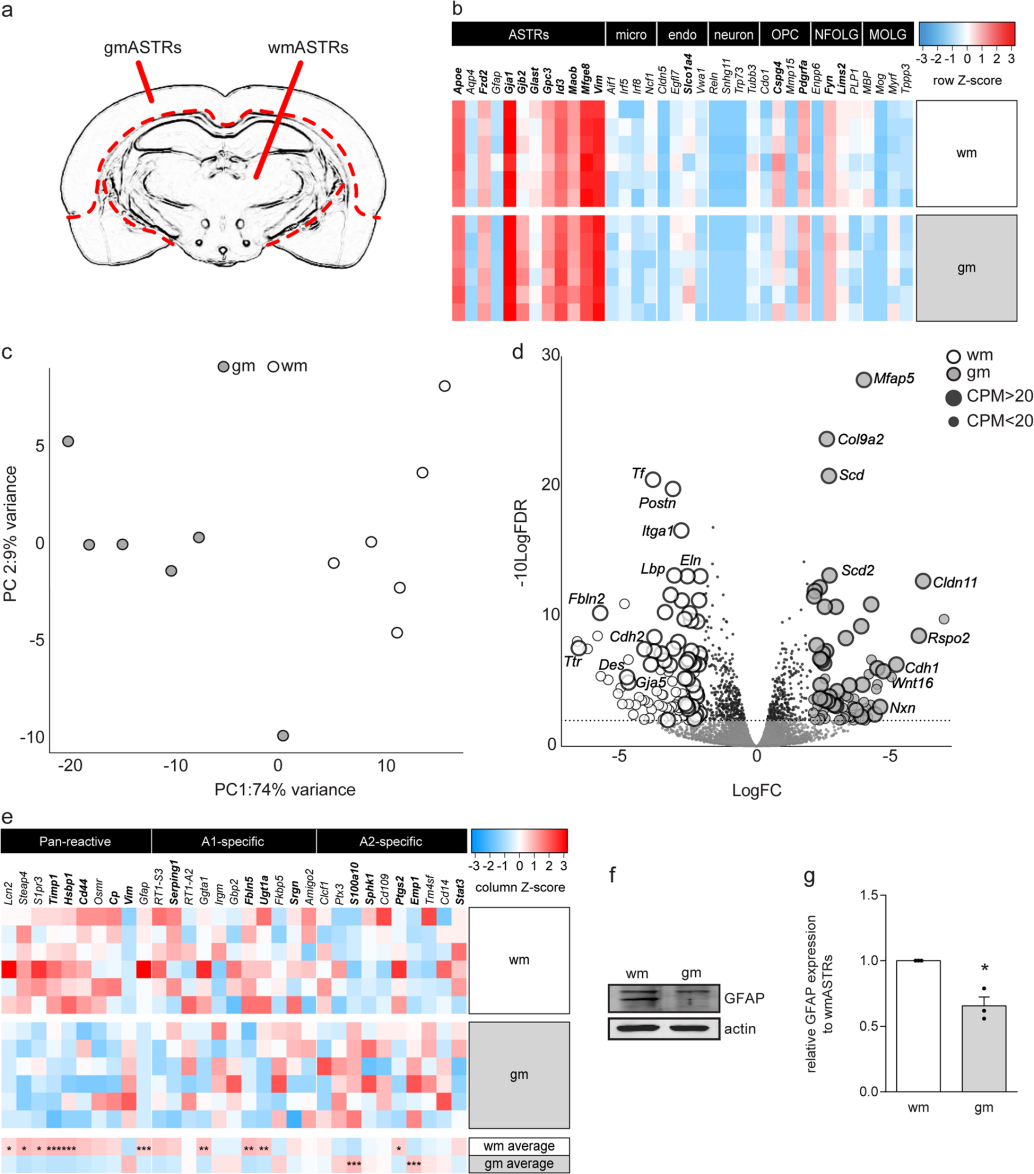
Primary adult wmASTRs and gmASTRs are transcriptionally heterogeneous. RNA from six independent cell culture preparations of adult grey matter astrocytes (gmASTRs) and adult white matter ASTRs (wmASTRs), was subjected to 3′-RNA sequencing. **a** Schematic representation of the dissected areas. **b** Heatmap of the logarithm of the gene count per million counts + 1 (log(CPM+1)) of genes specific for ASTRs, microglia (micro), endothelial cells (endo), neurons (neuron), oligodendrocyte progenitor cells (OPCs), newly formed oligodendrocytes (nfOLGs)-, and mature oligodendrocytes (mOLGs). Row Z-score represents relative expression of cell type-specific genes compared to each other within one biological sample. Genes with a CPM > 20 are depicted in bold. Note that most ASTR genes are highly expressed within different batches compared to other cell type-specific genes. **c** Principal component analysis (PCA) indicates that 74% of the variation between the samples is explained by their regional origin. **d** A volcano plot displaying all genes. The white dots depict genes more abundantly expressed in wmASTRs, and grey dots display genes more abundantly expressed in gmASTRs (absolute logarithm of fold change (logFC) > 2, FDR < 0.01). The small black dots are all remaining genes that are identified but not differentially expressed. **e** Heatmap of pan-reactive-, A1-, and A2-specific ASTR markers. Column Z-score represents the relative expression of genes between different samples. Genes with a CPM > 20 are depicted in bold (*FDR < 0.05, **FDR < 0.01, ***FDR < 0.001). Note that several pan-reactive and A1-ASTR-specific genes are more expressed in wmASTRs, while some A2-ASTR-specific genes are more expressed in gmASTRs. **f**, **g** Western blot analysis of GFAP. A representative image is shown in **f**, and quantification of three independent ASTR culture preparations (black dots) in **g**. Bars represent the relative mean to wmASTRs, which were set to 1 in each independent experiment. Error bar indicates the standard error of the mean. Statistical analyses are performed using column statistics with a one-sample *t* test (*p < 0.05) to assess differences between gmASTRs and wmASTRs. Note that GFAP expression is higher in wmASTRs (*p* = 0.038)

Previous findings indicate that remyelination is more efficient in GM lesions than in WM lesions [37, 38, 40, 41]. OLGs depend on support from ASTRs to remyelinate denuded axons [4–8], and as gmASTRs and wmASTRs are morphologically and functionally different [27, 28, 31, 32], we aimed to address whether gmASTRs and wmASTRs distinctly modulate myelination. As cultured neonatal gmASTRs are still considered as (reactive) ASTR progenitors [58, 59], we used an in vitro culture model of ASTRs derived from young adult rat brains [51]. These primary ASTRs exhibit properties like the expression of ASTR markers, glutamate uptake, and responses to injury, that represent their properties in the adult brain [51]. To assess whether primary adult gmASTRs and wmASTRs differ, the transcriptional profiles of ASTRs from the cerebral cortex (GM, referred to as gmASTRs) and non-cortical brain regions (mainly WM, referred to as wmASTRs, but including some deep gmASTRs) were compared after 20 days in culture (Fig. 1a). Analysis of CNS cell type-specific gene expression revealed a high abundance of transcripts of most ASTR-specific genes (e.g., *Vim*, *Mfeg8*, *Gja1*), and relatively low expression of OPC- (e.g., *Cspg4*, *Pdgra*), newly formed OLG- (e.g., *Enpp6*), and mature OLG-specific (e.g., *Mbp*, *Mog*) genes. Genes typically expressed by microglia (e.g. *Irf8*, *Af1*, *Ncf1*), endothelial cells (e.g., *Cldn5*, *Egfl7*, *Vwa1*), and neurons (e.g., *Tubb3*, *Reln*, *Trp73*) also have a low abundance (Fig. 1b). These data indicate that both adult gmASTRs and wmASTRs cultures consisted of highly pure astrocytes. Principal component analysis (PCA) indicated that 74% of the variance between the samples was explained by region of origin of the ASTRs (Fig. 1c), indicating that primary adult gmASTR and wmASTRs were transcriptionally different. Between gmASTR and wmASTRs, 857 genes were differentially expressed (FDR < 0.01), and 183 genes with a logFC > 2. Of these genes, 75 genes were more abundantly expressed in gmASTRs (additional file 1) and 108 genes in wmASTRs (additional file 1). Genes that were more abundantly expressed in wmASTRs include several ECM-related genes (*Fbln2*, *Eln*, *Postn*, *Itga1*), while in gmASTRs, genes involved in lipogenesis (*Scd*, *Scd2*) and Wnt signaling (*Rspo2*, *Wnt16*) were more abundantly expressed. Strikingly, two different members of the cadherin family are opposingly differentially expressed; *Cdh2* encoding for N-cadherin was more abundantly expressed in wmASTRs, while transcripts for *Cdh1* encoding for E-cadherin were more present in gmASTRs (Fig. 1d). We next compared the transcription profiles with the list of marker genes characteristic for reactive ASTRs in general (pan-reactive genes) and marker genes for neurotoxic A1-ASTRs and neuroprotective A2-ASTRs as defined by Liddelow et al [22]. Most pan-reactive ASTR marker genes were more abundantly expressed in primary adult wmASTRs than in primary adult gmASTRs (Fig. 1e, *Lcn2*, FDR = 0.021; *Gfap*, *Timp1*, and *Hsbp1* FDR < 0.001). Notably, transcript levels for some A1-ASTR-associated genes were more present in wmASTRs (*Ggta1* FDR = 0.002; *Fbln5* FDR = 0.005; *Ugt1a* FDR = 0.008), while transcripts of two A2-ASTR-associated genes (*S100a10* and *Emp1* FDR < 0.001) were more abundant in gmASTRs (Fig. 1e). Although *Gfap* mRNA expression was relatively low in both types of ASTRs, GFAP protein was present at detectable levels (Fig. 1f). Consistent with *Gfap* mRNA levels (Fig. 1e), GFAP protein levels were significantly higher in wmASTRs compared to gmASTRs (Fig. 1g), most likely due the low expression of a GFAP splice variant by gmASTRs (Fig. 1f). Notably, although previous profiling studies demonstrated that primary neonatal gmASTRs cultured in the presence of serum induces the expression of a specific set of genes [59], none of these serum-induced genes were expressed by our primary adult ASTRs. Hence, primary adult wmASTRs and gmASTRs were heterogeneous at the transcription level even upon prolonged time in culture. To assess whether gmASTRs and wmASTRs distinctly modulate myelination efficiency, their ability to regulate in vitro myelination was examined next.

### Primary adult wmASTRs are less supportive to myelination than primary adult gmASTRs

**Fig. 2:**
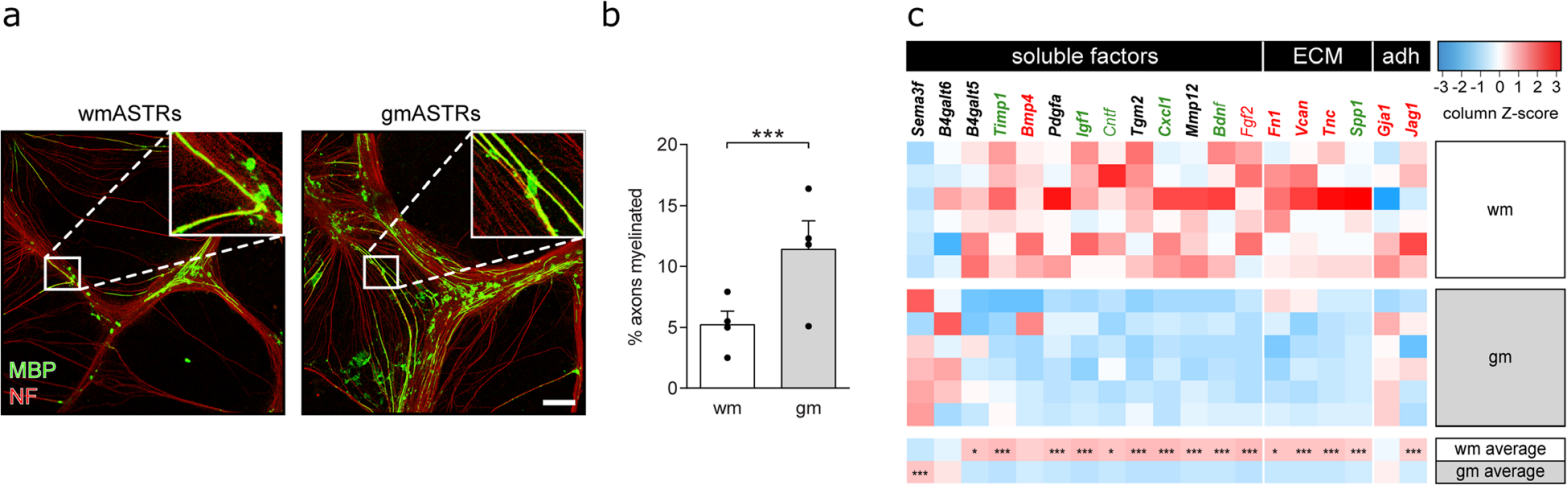
WmASTRs are less supportive to in vitro myelination. **a**, **b** In vitro myelination cultures that depend on a feeding layer of astrocytes (ASTRs) are stained for myelin basic protein (MBP, green) and neurofilament-H (NF, red), a myelin and axonal marker, respectively. Representative images of myelinating cultures on either adult white matter (wm) ASTRs or adult grey matter (gm) ASTRs are shown in **a** and quantification of the percentage of myelinated axons of four independent culture preparations and four independent ASTR culture preparations (black dots) in **b**. Bars represent the % of myelinated axons. Error bars represent standard error of the mean. Note that less axons are myelinated on a feeding layer of wmASTRs than on a feeding layer of gmASTRs. Scale bar is 25 μm. **c** RNA from six independent cell culture preparations of adult gmASTRs and wmASTRs was subjected to 3′-RNA sequencing. Heatmap with literature-based genes of positive and negative regulators of OPC proliferation and differentiation by gmASTRs and wmASTRs is shown. Column Z-score represents the relative expression of genes between different samples. Positive regulators of OPC differentiation are indicated in green; negative regulators in red (*FDR < 0.05, **FDR < 0.01, ***FDR < 0.001).

**Fig. 3:**
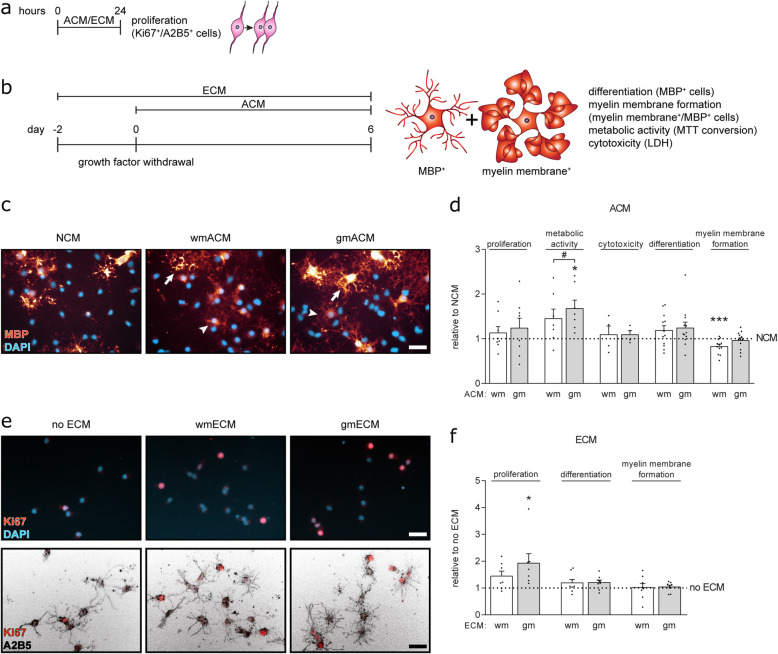
Secreted factors from wmASTRs inhibit myelin membrane formation. **a**–**f** Oligodendrocyte progenitors cells (OPCs) were cultured with PDGF-AA and FGF2 for 24 h to assess proliferation (% Ki67-positive cells of A2B5-positive cells, schematic representation in **a**), or differentiated into mature oligodendrocytes (OLGs) for 6 days after growth factor withdrawal to assess metabolic activity (MTT), cytotoxicity (LDH), differentiation (% MBP-positive cells), and myelin membrane formation (% myelin membranes formed by MBP-positive cells) (schematic representation in **b**). Assays were performed in the presence or absence of astrocyte (ASTR)-conditioned medium (ACM) or ASTR-derived extracellular matrix (ECM) coatings obtained from primary adult grey matter (gm) or white matter (wm) ASTRs. Representative images of MBP-positive OLGs (red, arrow indicates a MBP-positive cell with myelin membrane, arrowhead a MBP-positive cell without myelin membrane) in the presence of non-conditioned medium (NCM) or ACM are shown in **c**; representative images of proliferation on no ECM (PLL) or ECM coatings stained for Ki67 (red) and A2B5 (black) are shown in **d**. Quantification of assays of 4–12 independent cell culture preparations (black dots) with 4–12 different batches of ACM (**e**) and ECM coatings (**f**) are shown. Bars represent the relative means to NCM (**e**) or no ECM (**f**), which were set to 1 in each independent experiment. Error bars indicate the standard error of the mean. Statistical analyses are performed using column statistics with a one-sample *t* test (*p < 0.05) to assess differences between treatments and control, while a paired two-sided *t* test (*p < 0.05) is used to examine differences between effects of gmACM or gmECM coatings versus respectively wmACM or wmECM coatings. Absolute values of NCM are for proliferation 37.5 ± 22.4%, cytotoxicity 25.9 ± 2.9%, differentiation 48.6 ± 17.6%, and myelin membrane formation 68.0 ± 14.4% and of no ECM for proliferation 17.8 ± 4.8%, differentiation 33.3 ± 4.7%, and myelin membrane formation 65.3 ± 3.8%. Note that upon exposure to wmACM, myelin membrane formation is decreased compared to NCM (*p* = 0.005), while metabolic activity is increased upon gmACM treatment compared to NCM (*p = 0.016) and wmACM treatment (^#^*p* = 0.033) (**c**, **d**). In addition, OPC proliferation is higher on gmECM coatings than on no ECM (*p* = 0.041) (**e**, **f**). Scale bars are 25 μm

To study the role of gmASTRs and wmASTRs in myelination efficiency, we made use of an in vitro myelinating system of total embryonic spinal cord cultures that only develop on an ASTR feeding layer [57]. Myelination efficiency was assessed by double labeling of myelin and neurons, using myelin basic protein (MBP) as a marker for myelin, and neurofilament-H (NF) as a marker for neurons. While the axonal density was not significantly different (gmASTRs 8.3 ± 2.4%, wmASTRs 7.1 ± 1.2%), the percentage of myelinated axons was higher on a feeding layer of gmASTRs than on a feeding layer of wmASTRs (Fig. 2a, b, *p* = 0.016). This difference in their ability to support in vitro myelination is either a result of wmASTR-derived myelination-inhibiting or gmASTR-derived myelination-stimulating signals. ASTRs can signal to OPCs via secreted soluble factors, such as growth factors and cytokines, via insoluble factors, such as ECM proteins, and via adhesive interactions. The transcriptional profiles uncovered that wmASTRs expressed more transcripts of both positive and negative regulators of OPC proliferation (*Pdgfra*, *Fgf2* [52, 60, 61]) and OPC maturation (*Bmp4*, *Cntf*, *Timp1*, *Fn*, *Vcan*, *Tn*c, *Jag1*, *Igf1*, *Gja1, Tgm2* [62–71]) than gmASTRs (Fig. 2c). To determine whether wmASTRs and gmASTRs differentially support OPC behavior by a difference in secreted factors, the effect of ASTR-conditioned medium (ACM) of either ASTR on OPC proliferation, metabolic activity, and/or maturation was examined (Fig. 3a–c). OPC proliferation, as assessed by the number of Ki67-positive cells of A2B5-positive OPCs, was similar when OPCs were exposed to gmACM, wmACM, and non-conditioned medium (NCM) (Fig. 3d). After 6 days of differentiation, the metabolic activity, as assessed by an MTT assay, was increased upon exposure to gmACM compared to both wmACM and NCM (Fig. 3d, gmACM versus NCM *p* = 0.016; gmACM versus wmACM *p* = 0.033). Remarkably, ACM from neither ASTR was toxic to the cells (Fig. 3d), despite wmASTRs more abundantly expressing specific genes for neurotoxic A1-ASTRs (Fig. 1e). In addition, while the percentage of MBP-positive OLGs, reflecting their differentiation, remained similar, the percentage of MBP-positive OLGs that elaborate myelin membranes was significantly decreased in the presence of wmACM compared to NCM (Fig. 3c, d, *p* = 0.005). Exposure to gmACM did not affect OPC differentiation or myelin membrane formation (Fig. 3d). To examine the effect of ASTR-derived ECM on OPC behavior, OPCs were cultured on coatings of gmECM or wmECM. OPC proliferation was significantly increased when OPCs were plated on a gmECM coating compared to an inert PLL-coating (no ECM, Fig. 3e, f, *p* = 0.041). OPC proliferation was not affected by a wmECM coating (Fig. 3d–f). OPC differentiation and myelin membrane formation were not altered when plated on either gmECM or wmECM coatings compared to a PLL-coating (Fig. 3f). Hence, these findings revealed that wmASTRs were less supportive to in vitro myelination than gmASTRs, which may be the result of an inhibitory effect of wmASTR-derived secreted factor(s) that preclude myelin membrane formation and/or a stimulatory effect of gmASTR-deposited ECM on OPC proliferation. To obtain more insight into the gene networks that drive the distinct regulation of myelination by gmASTRs and wmASTRs, a weighted gene co-expression network analysis (WGCNA) was performed.

### Gene co-expression networks differ between primary adult wmASTRs and gmASTRs

**Fig. 4:**
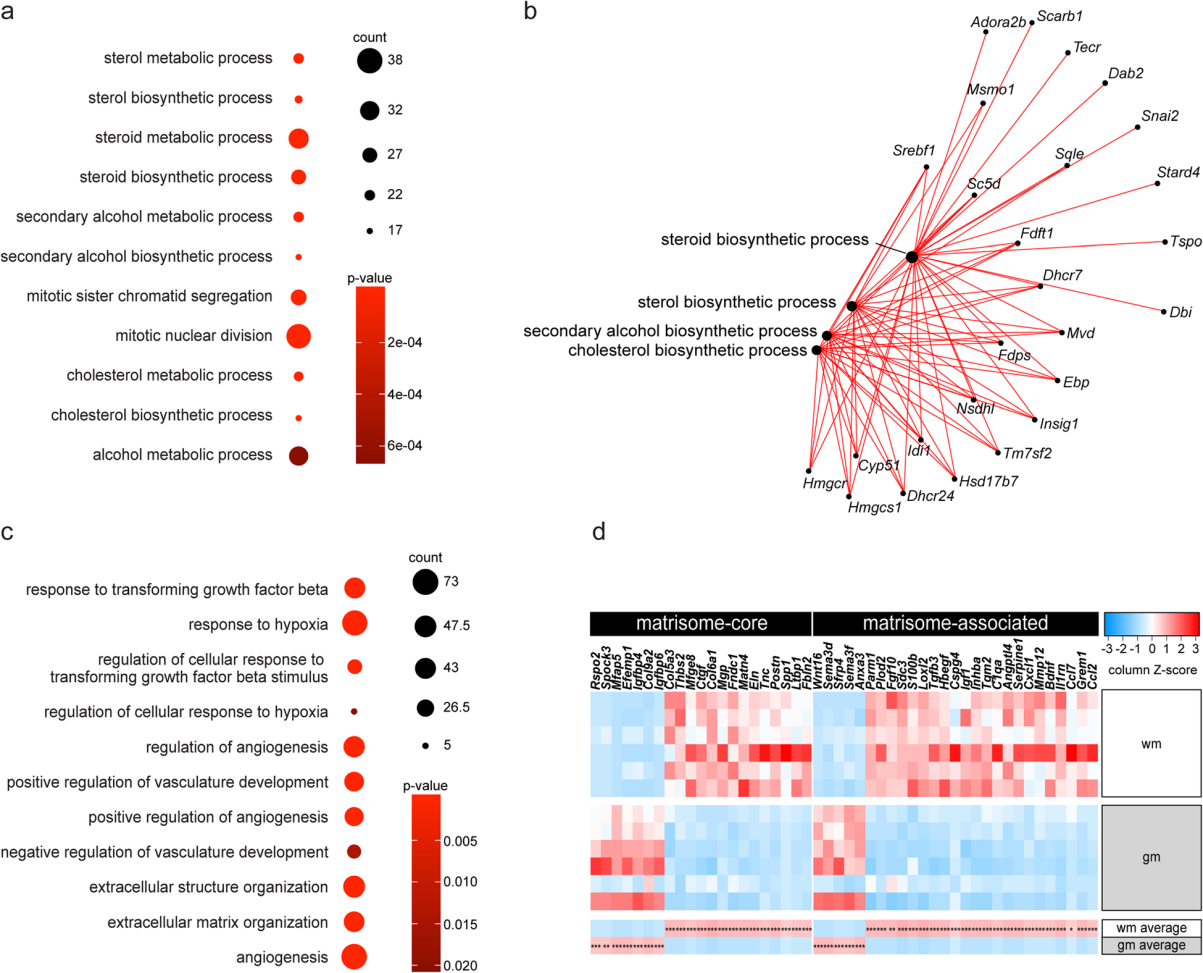
Gene co-expression networks differ between primary adult wmASTRs and gmASTRs. RNA from six independent cell culture preparations of adult grey matter astrocytes (gmASTRs) and adult white matter ASTRs (wmASTRs) was subjected to 3′-RNA sequencing. A weighted gene co-expression network analysis revealed that the MEroyalblue module positively correlated with gmASTRs and negatively with wmASTRs and that the MEdarkgrey module negatively correlated with gmASTRs and positively with wmASTRs (Fig. S1, Additional file 2). **a** Gene ontology (GO) annotation of the genes in the MEroyalblue module visualized in a dot plot. The color of the dot represents the enrichment score (*p* value), the size of the dot the gene count. Note that genes related to functions as “cell division” and “cholesterol biosynthesis” are enriched in the MEroyalblue module. **b** Visualization of the interaction network of the genes present in the four indicated GO categories (black dots) from the MEroyalblue cluster. Note that some genes are shared between different GO terms. **c** GO annotation of the genes in the MEdarkgrey module visualized in a dot plot. The color of the dot represents the enrichment score (*p* value), the size of the dot the gene count. Note that genes related to functions as “response to hypoxia” “extracellular matrix organization” are enriched int the MEdarkgrey module. **d** Heatmap of the extracellular matrix proteome or “matrisome”-core and -associated genes with a CPM > 20, an absolute logFC > 2, and an FDR < 0.05 by gmASTRs and wmASTRs. Column Z-score represents the relative expression of genes between different samples. Note that more genes encoding for matrisome-core and -associated genes are more abundantly expressed in wmASTRs (*FDR < 0.05, **FDR < 0.01, ***FDR < 0.001)

WGCNA is a powerful method that determines correlated changes in gene expression allowing for the identification of modules of similarly expressed gene networks that represent biological functions and regulatory mechanisms [72]. WGCNA analysis identified 14 modules of highly connected genes with similar expression pattern (Fig. S1a, b, additional file 2). The correlation between each Module Eigengene (ME) and the trait region (gmASTRs and wmASTRs) revealed that the MEroyalblue and MEdarkgrey modules were significantly and differentially correlated with region across the biological replicates of gmASTR and wmASTR cell culture preparations (Fig. S1b-d, additional file 2). Module MEroyalblue, consisting of 844 genes, was positively correlated with gmASTRs (*p* < 0.001) and negatively correlated with wmASTRs (Fig. S1b, c, additional file 2). To retrieve the biological processes the genes of this module are involved in, gene ontology (GO) annotation analysis was performed. Many genes represented in the MEroyalblue module were related to “cell division” (Fig. 4a). Additionally, genes that are enriched in the MEroyalblue module were related to GO terms “sterol biosynthetic process,” “steroid biosynthetic process,” “cholesterol biosynthetic process,” and “secondary alcohol biosynthetic process” (Fig. 4a). An interaction network of the genes in these biosynthetic processes demonstrated that some of these genes are shared between multiple GO terms (Fig. 4b). A heatmap of relevant sterol, steroid, and cholesterol biosynthesis genes (Fig. S2, additional file 3) illustrates a higher expression of these genes in gmASTRs than in wmASTRs, which predicts that there is more cholesterol biosynthesis in primary adult gmASTRs. Notably, ASTRs supply cholesterol to OLGs, which supports myelination [4, 7, 73].

The other significantly different module, MEdarkgrey, consists of 1110 genes and positively correlated with wmASTRs (*p* < 0.001) and negatively with gmASTRs (Fig. S1b, d, additional file 2). GO terms related to this module included processes as “ECM formation and modification,” “atherosclerosis,” “hypoxia,” and “vascularization” (Fig. 4c). Of interest, transient ECM remodeling plays an important role in successful remyelination [5, 57, 74, 75]. The extracellular matrix proteome, or the “matrisome” consists of core and associated proteins [76] and a heatmap of abundantly expressed matrisome-core and matrisome-associated genes that differed significantly (FDR < 0.05) (Fig. 4d) illustrates that these genes are more abundantly expressed in wmASTRs than in gmASTRs. These more abundant genes encode for ECM-core proteins (e.g., *Mfeg8*, *Ctgf*, *Spp1*, *Ltbp1*) that are mainly matricellular proteins, and ECM-affiliated proteins (e.g., *Bdnf*, *Hbegf*, *Mmp1*2, *Ccl2*, *Ccl7*), which are proteins that regulate ECM remodeling or secreted factors that associated with the ECM. Notably, transcripts of *Sema3f*, an OPC attractant, were more present in gmASTRs than in wmASTRs (Fig. 4d). Several ECM genes that were more abundantly expressed in primary adult wmASTRs were associated with pathways previously implicated in OPC maturation and/or remyelination failure (*Ctgf*, *Tnc*, *Spp1* [21, 67, 77–79]) and/or expressed in reactive ASTRs in WM MS lesions (*Mfeg8*, *Tnc*, *Spp1* [34, 77, 80, 81]), and may relate to the more reactive ASTR profile of primary adult wmASTRs (Fig. 1). Hence, the regulatory mechanisms that may account for the higher in vitro myelination potential of OLGs in the presence of gmASTRs, are more cholesterol biosynthesis in gmASTRs and increased deposition of matrisome constituents by wmASTRs. The different identities of gmASTRs and wmASTRs may result in distinct and specific responses toward inflammatory mediators that are increased upon injury. As demyelination involves innate immune activation, and as TLR3 and TLR4 are prominently present on reactive ASTRs in WM MS lesions [13, 49], we next examined whether gmASTRs and wmASTRs may respond differently to TLR3 and/or TLR4 agonists and as a consequence distinctly modulate OPC behavior.

### Adult gmASTRs and wmASTRs responding to TLR4 agonist LPS decrease myelin membrane formation

**Fig. 5:**
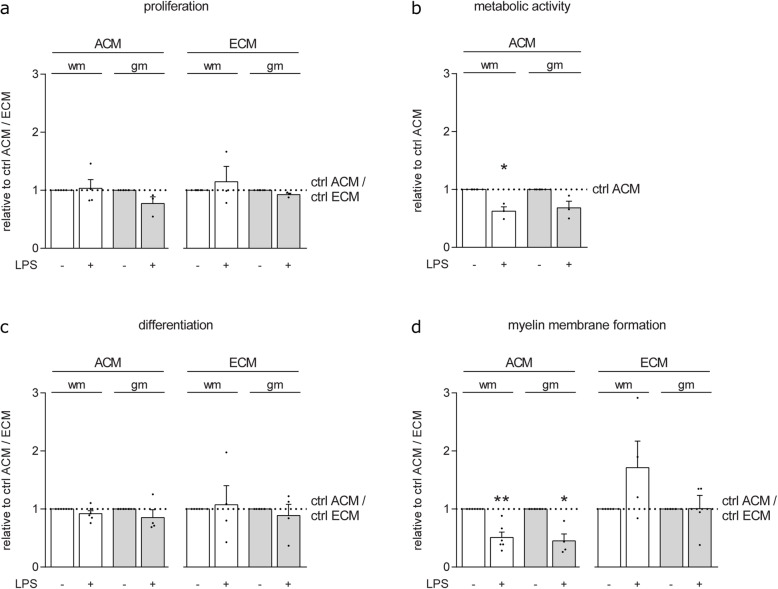
Secreted factors from primary adult ASTRs responding to TLR4 agonist LPS decrease myelin membrane formation*.* **a**–**d** Primary adult grey matter astrocytes (gmASTRs) and white matter ASTRs (wmASTRs) were cultured for 24 h in the presence or absence of TLR4 agonist LPS, after which conditioned medium (ACM) was collected in the next 24 h. For extracellular matrix proteins (ECM), ASTRs were cultured for 48 h in the presence or absence of LPS after which the cells were lysed, ECM was collected, and used as a coating. Oligodendrocyte progenitor cells (OPCs) were cultured in the presence of ACM or on ECM coatings, for 1 day in the presence of PDGF-AA and FGF-2 to assess proliferation (**a**, % Ki67-positive cells of A2B5-positive cells), or differentiated for six 6 days after growth factor withdrawal to assess metabolic activity (**b**, MTT), differentiation (**c**, % MBP-positive cells of total cells), and myelin membrane formation (**d**, % myelin membranes formed by MBP-positive cells). Bars represent relative means to their respective untreated control ACM or ECM coatings, which were set to 1 in each independent experiment. Individual data points (black dots) represent independent OPC culture preparations with three to five different batches of ACM or ECM. Error bars indicate standard error of the mean. Statistical analyses are performed using column statistics with a one-sample *t* test (*p < 0.05) to assess differences with untreated control ACM or ECM coatings and with an unpaired two-sided *t* test (not significant) to assess differences between response to ACM or ECM coatings of LPS-treated gmASTRs compared to ACM or ECM coatings of LPS-treated wmASTRs. Absolute values are 34.3 ± 17.4% proliferation, 55.1 ± 19.1% differentiation, and 56.8 ± 17.7% myelin membrane formation with control wmACM; 39.6 ± 13.5% proliferation, 61.6 ± 22.2% differentiation, and 67.4 ± 20.3% myelin membrane formation with control gmACM; 31.4 ± 12.5% proliferation, 51.9 ± 18.6% differentiation, and 56.7 ± 22.3% myelin membrane formation on control wmECM coatings and 41.0 ± 1.7% proliferation, 44.8 ± 15.1% differentiation, and 51.5 ± 12.5% myelin membrane formation on control gmECM coatings. Note that myelin membrane formation is decreased in the presence of ACM of both LPS-treated wmASTRs and gmASTRs (wmACM *p* = 0.003, gmACM *p* = 0.020)

As TLR3 and TLR4 agonists interfered with the development of neurons, OPCs, and OLGs in the in vitro myelinating cultures, we were not able to examine the role of ASTR reactivity on in vitro myelination. Therefore, to assess whether gmASTRs and wmASTRs differently respond to TLR4 ligands, the effect of TLR4 agonist LPS on primary ASTRs and their ability to modulate OPC behavior via secreted factors (ACM) or coatings of deposited ECM components was determined. Secreted factors from LPS-treated wmASTRs and gmASTRs did not significantly change OPC proliferation (Fig. 5a) and differentiation (Fig. 5c). Secreted factors from both LPS-treated gmASTRs and wmASTRs markedly inhibited myelin membrane formation by 40–50% compared to secreted factors from their respective untreated ASTRs (Fig. 5d, gmACM *p* = 0.020, wmACM *p* = 0.003). This is accompanied by a significant decrease in metabolic activity by 30–40% upon wmACM treatment (Fig. 5b wmACM *p* = 0.039), and a reproducible, but not significant decrease upon gmACM treatment (Fig. 5b). ECM coatings of LPS-treated gmASTRs or wmASTRs did not significantly alter OPC proliferation (Fig. 5a), differentiation (Fig. 5b), or myelin membrane formation (Fig. 5c). Hence, our findings demonstrate that LPS-treated wmASTRs and gmASTRs similarly modulate OPC behavior by secreting factors that reduced the percentage of MBP-positive OLGs that form myelin membranes.

### Adult gmASTRs and wmASTRs responding to TLR3 agonist Poly(I:C) distinctly modulate OPC proliferation and myelin membrane formation

**Fig. 6:**
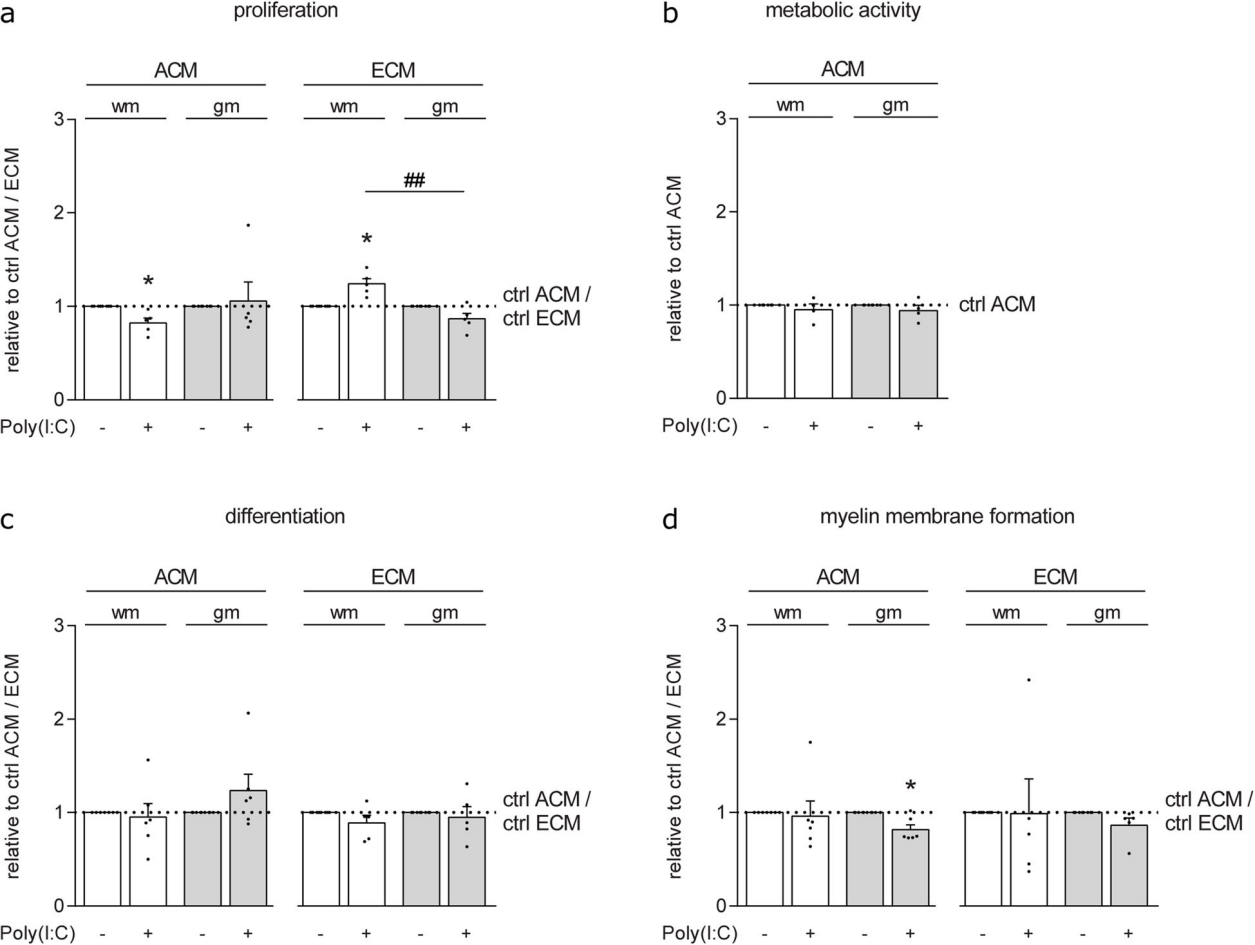
Primary adult gmASTRs and wmASTRs responding to TLR3 agonist Poly(I:C) distinctly modulate OPC behavior*.* **a**–**d** Primary adult grey matter astrocytes (gmASTRs) and white matter ASTRs (wmASTRs) were cultured for 24 h in the presence or absence of TLR3 agonist Poly(I:C), after which conditioned medium (ACM) was collected in the next 24 h. For extracellular matrix proteins (ECM), ASTRs were cultured for 48 h in the presence or absence of Poly(I:C) after which the cells were lysed, ECM was collected and used as a coating. Oligodendrocyte progenitor cells (OPCs) were cultured in the presence of ACM or on ECM coatings, for 1 day in the presence of PDGF-AA and FGF-2 to assess proliferation (**a**, % Ki67-positive cells of A2B5-positive cells), or differentiated for six days after growth factor withdrawal to assess metabolic activity (**b**, MTT), differentiation (**c**, % MBP-positive cells of total cells), and myelin membrane formation (**d,** % myelin membranes formed by MBP-positive cells). Bars represent relative means to their respective untreated control ACM or ECM coatings, which were set to 1 in each independent experiment. Individual data points (black dots) represent independent OPC culture preparations with three to five different batches of ACM or ECM. Error bars indicate standard error of the mean. Statistical analyses are performed using column statistics with a one-sample *t* test (*p < 0.05) to assess differences with untreated control ACM or ECM coatings, and with an unpaired two-sided *t* test (^##^*p* < 0.01) to assess differences between response to ACM or ECM coatings of Poly(I:C)-treated gmASTRs and ACM or ECM coatings of Poly(:C)-treated wmASTRs Absolute values are 42.5 ± 13.9% proliferation, 34.9 ± 7.6% differentiation, and 58.4 ± 9.1% myelin membrane formation with control wmACM; 38.4 ± 14.1% proliferation, 35.3 ± 12.2% differentiation, and 67.5 ± 10.1% myelin membrane formation with control gmACM; 21.6 ± 4.0% proliferation, 31.8 ± 5.7% differentiation, and 81.1 ± 10.1% myelin membrane formation on control wmECM coatings and 31.4 ± 5.8% proliferation, 37.5 ± 5.3% differentiation, and 75.8 ± 18.6% myelin membrane formation on control gmECM coatings. Note that OPC proliferation is decreased in the presence of ACM of Poly(I:C)-treated wmASTRs (^*^p = 0.027) and increased on ECM coatings of Poly(I:C)-treated wmASTRs (^*^p = 0.012), but not on ECM-coatings of gmASTRs (^##^*p* = 0.0016 compared to ECM from Poly(I:C)-treated wmASTRs), while myelin membrane formation is decreased in the presence of ACM of Poly(I:C)-treated gmASTRs (*p* = 0.016)

To assess whether gmASTRs and wmASTRs differently respond to TLR3 ligands, the effect of TLR3 agonist Poly(I:C) on ASTRs, and their ability to modulate OPC behavior was determined next. Secreted factors from Poly(I:C)-treated wmASTRs, but not from Poly(I:C)-treated gmASTRs, decreased OPC proliferation by approximately 18% compared to their respective untreated control ASTRs (Fig. 6a, *p* = 0.027). In contrast, OPC proliferation was increased when plated on ECM coatings of Poly(I:C)-treated wmASTRs compared to ECM coatings of untreated control wmASTRs (Fig. 6a, *p* = 0.012) and ECM coatings of Poly(I:C)-treated gmASTRs (Fig. 6a, *p* = 0.002). ACM of Poly(I:C)-treated gmASTRs reproducibly and significantly reduced myelin membrane formation by approx. 20% compared to untreated gmACM (Fig. 6d, *p* = 0.016). By contrast, myelin membrane formation was unchanged when OPCs were exposed to ACM of Poly(I:C)-treated wmASTRs (Fig. 5d). Secreted factors or ECM coatings from Poly(I:C)-treated gmASTRs or wmASTRs hardly affected the metabolic activity of OLGs and OPC differentiation compared to secreted factors or ECM coatings from their respective untreated control ASTRs (Fig. 6b, c). These data demonstrate that Poly(I:C) treatment of primary adult gmASTR and wmASTRs results in a modest but distinct modulation of OPC proliferation and myelin membrane formation. As secreted factors from Poly(I:C)-treated gmASTRs reduced myelin membrane formation, this may indicate that Poly(I:C)-treated gmASTRs are less supportive to myelination than untreated gmASTRs.

## Discussion

ASTRs support myelination during development and are indispensable for successful remyelination upon demyelination [2, 3, 17]. As remyelination is more efficient in GM lesions than in WM (MS) lesions [37, 38, 40, 41, 82], we aimed here to address whether the distinct modulation of myelination is a reflection of heterogeneity between gmASTRs and wmASTRs. Our findings demonstrate that primary adult wmASTRs were less supportive toward in vitro myelination than primary adult gmASTRs. Transcriptional profiling demonstrated that even after prolonged time in culture, adult gmASTRs and adult wmASTRs have a distinct transcriptional profile, which may underlie their difference in modulating in vitro myelination. Moreover, when exposed to TLR4 agonist LPS, secreted factors of both gmASTRs and wmASTRs markedly inhibited myelin membrane formation, while TLR3 agonist Poly(I:C)-treated gmASTRs and wmASTRs elicited modest but distinct responses in OPC proliferation and myelin membrane formation. Hence, our findings indicate that pre-existing regional heterogeneity in ASTRs may contribute to differences in remyelination efficiency in demyelinated GM and WM.

Our findings demonstrate that more axons were myelinated on a feeding layer of primary adult gmASTRs than on a feeding layer of primary adult wmASTRs. As secreted factors from wmASTRs, but not gmASTRs, decreased membrane formation, it is tempting to suggest that wmASTRs were less supportive to myelination. The transcriptional data provided insight into differences in gene expression of signaling molecules in gmASTRs and wmASTRs that are relevant for myelination and remyelination. Analysis of literature-based modulators of OPC behavior revealed that wmASTRs were equipped with higher transcript levels of genes encoding for signaling molecules that either positively or negatively modulate OPC proliferation and differentiation [52, 60, 61]. These signaling molecules were soluble (*Bmp4*, *Fgf2*, *Cntf*, *Pdgfa* [52, 60, 61, 64, 65]), ECM-derived (*Fn1*, *Vcan*, *Tnc*, *Spp1* [62, 67, 71, 75, 77]) or adhesion-dependent (*Jag1*). Likely, it reflects the *net* effect of these factors that made wmASTRs less supportive to myelination than gmASTRs in our in vitro myelinating culture system. Also, wmASTRs were more reactive than gmASTRs, and expressed more transcripts of several A1-ASTR-specific genes, while A2-ASTRs-specific genes were more abundantly expressed in gmASTRs. This is consistent with a recent single-nucleus RNA sequencing study of human control brain tissue that demonstrated transcriptional heterogeneity between human gmASTRs and wmASTRs [29], including a more reactive profile of wmASTRs compared to gmASTRs [29]. Hence, our primary adult gmASTRs and wmASTRs maintain some properties that are also observed in GM and WM areas.

In addition to wmASTRs being less supportive, gmASTRs may promote in vitro myelination. ECM components deposited by gmASTRs increased OPC proliferation, and as a higher density of OPCs augments myelination [83], enhanced proliferation may contribute to the increased in vitro myelination on gmASTRs. Also, an unbiased gene co-expression network analysis identified a gene module, which positively correlated with gmASTRs and negatively with wmASTRs that contained genes relevant to cell division and cholesterol biosynthesis. Cholesterol is a major constituent of myelin membranes [7], and in the adult brain mainly synthesized by ASTRs [4, 84]. Cholesterol enhances OPC differentiation [7, 73] and is the only known indispensable integral lipid in myelin of which its availability is rate-limiting for myelin growth [7]. Therefore, gmASTRs may supply more cholesterol to developing OLGs, which benefits myelination [4, 7, 73, 85]. Indeed, we recently demonstrated that neonatal gmASTRs efflux more cholesterol than neonatal wmASTRs [85]. Also, genes involved in cholesterol biosynthesis are downregulated in MS wmASTRs compared to ASTRs in healthy control WM [33], which may limit remyelination in WM lesions.

WGCNA further revealed that a gene module containing genes encoding for proteins that are relevant for ECM production and modification, i.e., the matrisome [86] was significantly correlated with wmASTRs. ASTRs are known to play an essential role in transient ECM remodeling, which is important for successful remyelination upon toxin-induced demyelination [75, 87–89]. In addition, increased expression of ECM proteins usually marks ASTR reactivity [12, 90]. Therefore, the enhanced expression of genes that are related to the matrisome is likely part of the more reactive phenotype of wmASTRs. Most of the matrisome-core enriched genes were matricellular proteins (*Thbs2*, *Postn*, *Ctgf*, *Fbln2*, *Spp1*), which are non-structural ECM proteins that support matrix fibrillogenesis and/or that have important functions in tissue repair [91]. These proteins may both influence matrix formation and signals to OPCs by modulating cell functioning via interaction with cell-surface receptors and structural ECM proteins, such as fibronectin and proteoglycans that are present in demyelinated areas [75, 88]. Thus, the effect of ECM coatings on OPC behavior presented in the present study are likely underestimated, as ECM coatings do not reflect all properties of the ECM, such as the original topological ECM architecture and stiffness. Indeed, the stiffness of the ECM influences OPC behavior; a stiff matrix favors OPC proliferation and initial differentiation and myelination is supported by a relatively soft matrix [6]. Hence, a higher abundance of ECM-related genes in wmASTRs may contribute to less efficient remyelination in WM lesions compared to GM lesions. This is indeed observed in leukocortical MS lesions, where ECM protein hyaluronan, an inhibitor of OPC differentiation [92], and its receptor CD44, are significantly increased in the WM, but not in the GM part of leukocortical lesions [41]. Also, wmASTRs form more remyelination-impairing fibronectin aggregates than gmASTRs [93], and fibronectin expression is increased in marmoset EAE WM, but not GM lesions [94]. Thus, interference with the wmASTR-mediated role in ECM remodeling may prove a valuable target for the enhancement of remyelination in WM MS lesions.

Diversity between wmASTRs and gmASTRs was also observed in their modulation of OPC behavior in response to TLR3 agonist Poly(I:C). More specifically, secreted factors of Poly(I:C)-treated gmASTRs, but not of Poly(I:C)-treated wmASTRs, decreased myelin membrane formation compared to secreted factors from their respective untreated ASTRs. Previous studies revealed that exposure to Poly(I:C) in rodent ASTRs induced the expression of both pro-inflammatory mediators that are linked to A1-ASTRs, and inflammatory mediators that are secreted by A2-ASTRs [22, 95]. In line with this reasoning, Poly(I:C) may distinctly interfere with gmASTR and wmASTR reactive subtypes. ASTR reactivity is also reflected in alterations in ECM remodeling, and OPC proliferation in response to ECM coatings of Poly(I:C)-treated wmASTRs significantly differed from ECM coatings of Poly(I:C)-treated gmASTRs. Thus, intrinsic differences between gmASTRs and wmASTRs may control remyelination differently by reacting in a region-specific manner to demyelinating injury. This is consistent with changes in gene expression between ASTRs from different regions in an animal model for MS [33], as well as differential responses of ASTRs isolated from different regions in vitro [32]. On the other hand, secreted factors from both TLR4 agonist LPS-treated gmASTRs and wmASTRs reduced myelin membrane formation. Therefore, the ASTR responses in GM and WM lesions may not only depend on the pre-existing heterogeneity of gmASTRs and wmASTRs and their differential response to the same type of inflammatory mediator, but may also relate to the local inflammatory context, which is different in GM and WM lesions [19, 41, 46, 96, 97].

## Conclusions

Taken together, primary adult gmASTRs and wmASTRs are diverse cell types, and heterogeneous at the transcriptional and GFAP protein level. They differ in their ability to modulate in vitro myelination and in their response to TLR3 agonists. As wmASTRs are intrinsically more reactive and less supportive to in vitro myelination, this may contribute to the more efficient remyelination observed in GM lesions compared to WM lesions. Identification of secreted or ECM-related factors that differentially modulate remyelination efficiency may identify new region-specific ASTR-targeted therapies for MS. In addition, validation of therapeutic tools for remyelination should take potential differences between gmASTRs and wmASTRs into account.

## Supplementary Information

**Additional file 1.** List of genes with CPMs, fold changes and p-values.

**Additional file 2: Figure S1.** Weighted gene co-expression network analysis on adult gmASTRs and wmASTRs. RNA from six independent cell culture preparations of adult grey matter astrocytes (gmASTRs) and adult white matter ASTRs (wmASTRs) was subjected to 3’-RNA sequencing and a weighted gene co-expression network analysis (WGCNA). **a** Cluster dendrogram of modules that contain similarly expressed genes. Each color at the bottom represents a defined module. **b** Heatmap of the correlation coefficient of the 14 modules of genes defined by WGCNA. The correlation between each Module Eigengene (ME) and the traits region and sample were calculated. The corresponding p-values is indicated. MEroyalblue and MEdarkgrey are significantly and differentially correlated with region. **c** The MEroyalblue gene module is positively correlated with gmASTRs and negatively with wmASTRs. **d** The MEdarkgrey gene module is positively correlated with wmASTRs and negatively with gmASTRs.

**Additional file 3: Figure S2.** Cholesterol biosynthesis genes are more abundantly expressed in grey matter astrocytes. RNA from six independent cell culture preparations of adult grey matter astrocytes (gmASTRs) and adult white matter ASTRs (wmASTRs) was subjected to 3’-RNA sequencing. Heatmap of identified sterol, steroid, and cholesterol biosynthesis genes in the MEroyalblue cluster from the weighted gene co-expression network analysis (WGCNA) in gmASTRs and wmASTRs is shown (Fig. 4a, b, Fig. S1, Additional file 2). Column Z-score represents the relative expression of genes between different samples. Genes with a CPM>20 are depicted in bold. Note that most genes encoding for cholesterol, steroid and sterol biosynthesis are more abundantly expressed in gmASTRs (*FDR<0.05, **FDR<0.01, ***FDR<0.001).

## Data Availability

The RNA-Seq data supporting the conclusions of this article are available in the Gene Expression Omnibus (GEO): GSE155866

## Abbreviations

ACM: Astrocyte-conditioned medium
ASTR: Astrocyte
BSA: Bovine serum albumin
CNS: Central nervous system
ECM: Extracellular matrix
FBS: Fetal bovine serum
GM: Grey matter
FGF2: Fibroblast growth factor-2
GO: Gene ontology
LDH: Lactate dehydrogenase
logFC: Logarithm of the fold change
LPS: Lipopolysaccharide
MBP: Myelin basic protein
MS: Multiple sclerosis
MTT: 3-(4,5-Dimethyl-2-thiazolyl)-2,5-diphenyl-2H-tetrazolium bromide
NCM: Non-conditioned medium
NF: Neurofilament-H
OPC: Oligodendrocyte progenitor cell
OLG: Oligodendrocyte
PCA: Principal component analysis
PFA: Paraformaldehyde
PLL: Poly-l-lysine
Poly(I:C): Polyinosine-polycytidylic acid
PVDF: Polyvinylidene fluoride
SEM: Standard error of the mean
TLR: Toll-like receptor
WGCNA: Weighted gene co-expression network analysis
WM: White matter
