# Supplementary figures and images for: Transcriptional heterogeneity between primary adult grey and white matter astrocytes underlie differences in modulation of in vitro myelination
Source: J Neuroinflammation. 2020 Dec 11;17:373. doi: 10.1186/s12974-020-02045-3 (PMC7733297; doi:10.1186/s12974-020-02045-3)

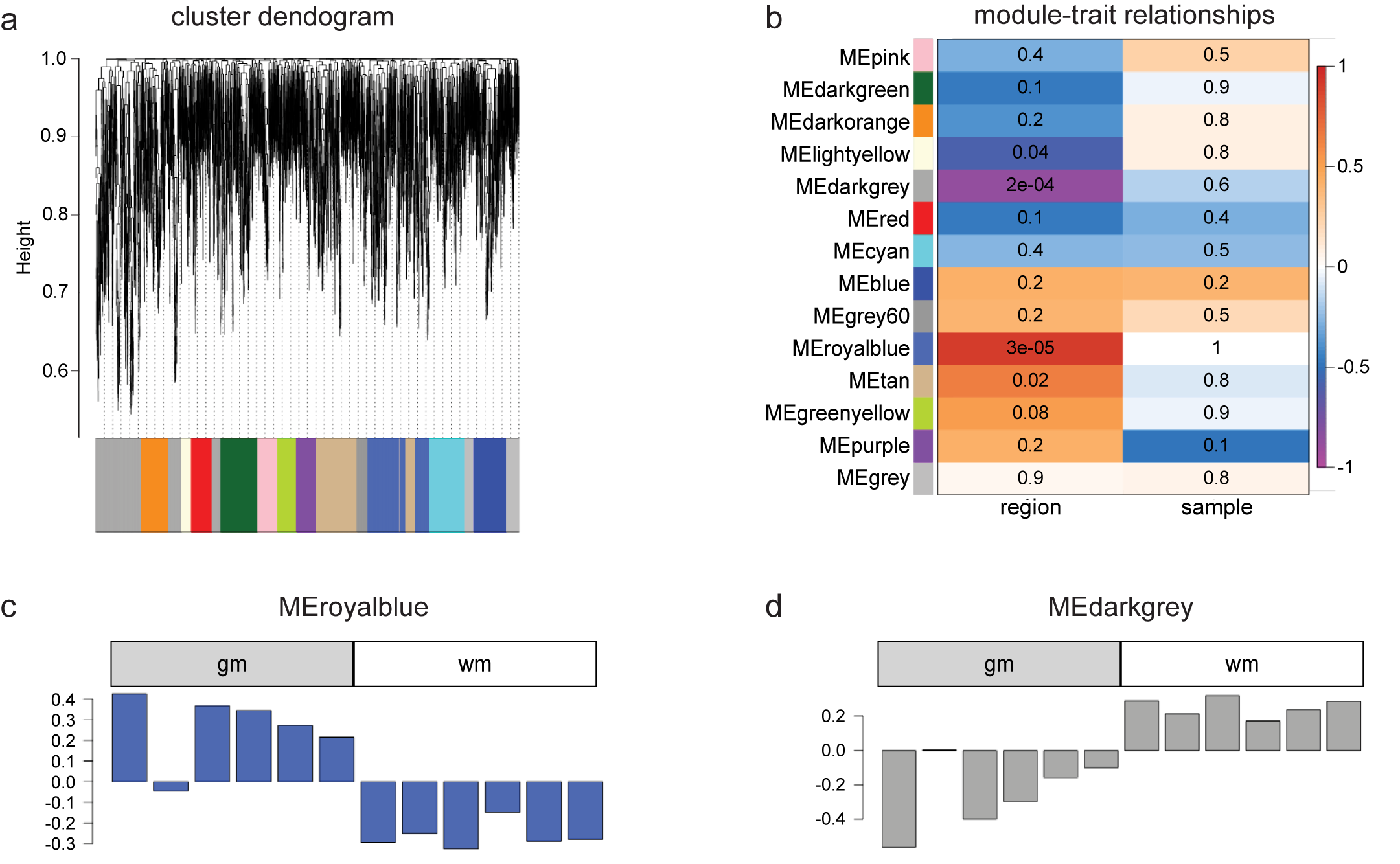

Supplement: Supplementary file 2 — Additional file 2: Figure S1. Weighted gene co-expression network analysis on adult gmASTRs and wmASTRs. RNA from six independent cell culture preparations of adult grey matter astrocytes (gmASTRs) and adult white matter ASTRs (wmASTRs) was subjected to 3’-RNA sequencing and a weighted gene co-expression network analysis (WGCNA). a Cluster dendrogram of modules that contain similarly expressed genes. Each color at the bottom represents a defined module. b Heatmap of the correlation coefficient of the 14 modules of genes defined by WGCNA. The correlation between each Module Eigengene (ME) and the traits region and sample were calculated. The corresponding p-values is indicated. MEroyalblue and MEdarkgrey are significantly and differentially correlated with region. c The MEroyalblue gene module is positively correlated with gmASTRs and negatively with wmASTRs. d The MEdarkgrey gene module is positively correlated with wmASTRs and negatively with gmASTRs. [file 12974_2020_2045_MOESM2_ESM.tif]

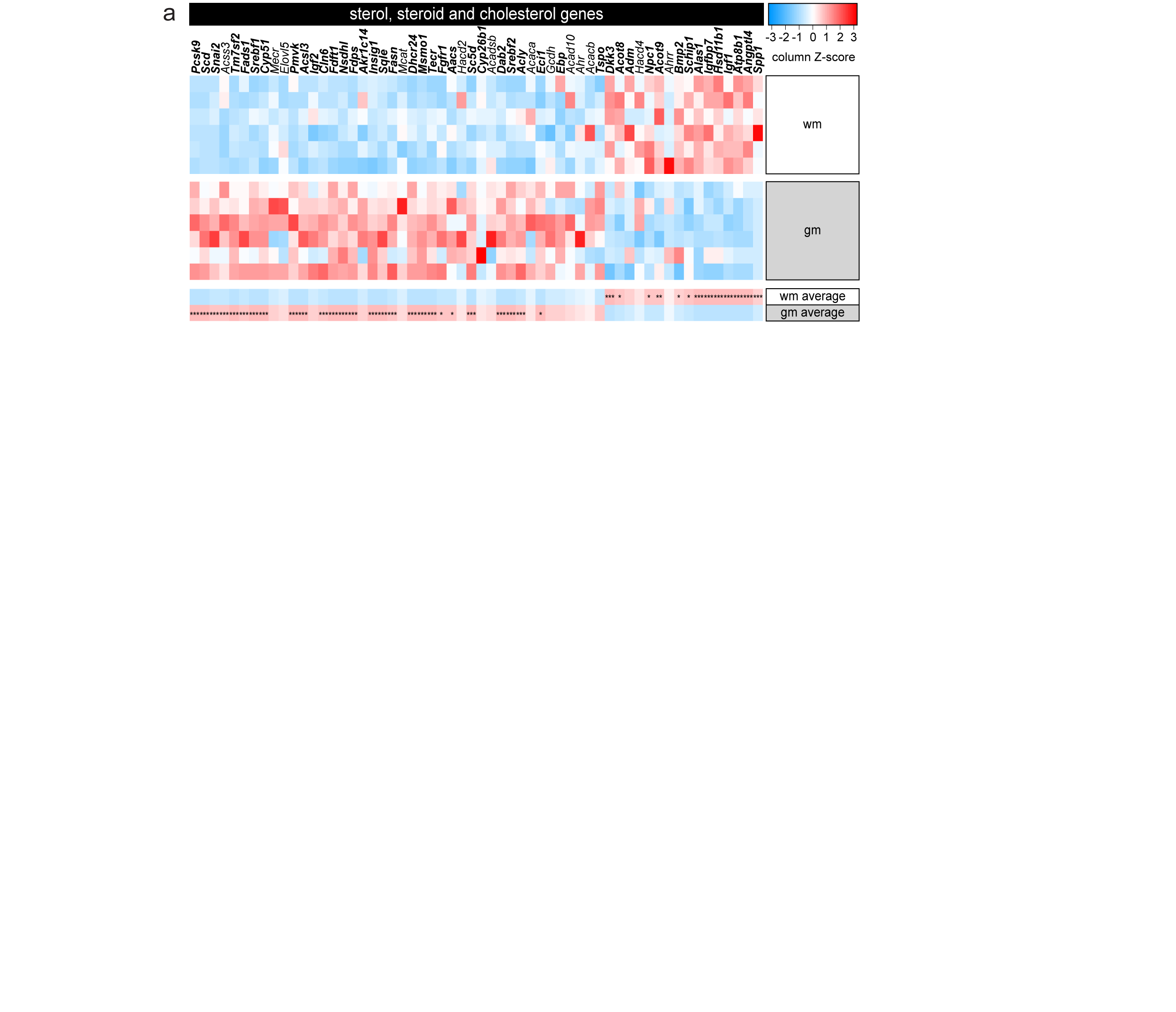

Supplement: Supplementary file 3 — Additional file 3: Figure S2. Cholesterol biosynthesis genes are more abundantly expressed in grey matter astrocytes. RNA from six independent cell culture preparations of adult grey matter astrocytes (gmASTRs) and adult white matter ASTRs (wmASTRs) was subjected to 3’-RNA sequencing. Heatmap of identified sterol, steroid, and cholesterol biosynthesis genes in the MEroyalblue cluster from the weighted gene co-expression network analysis (WGCNA) in gmASTRs and wmASTRs is shown (Fig. 4a, b, Fig. S1, Additional file 2). Column Z-score represents the relative expression of genes between different samples. Genes with a CPM>20 are depicted in bold. Note that most genes encoding for cholesterol, steroid and sterol biosynthesis are more abundantly expressed in gmASTRs (*FDR<0.05, **FDR<0.01, ***FDR<0.001). [file 12974_2020_2045_MOESM3_ESM.tif]
